# Gas chromatography techniques to evaluate the hydrogen permeation characteristics in rubber: ethylene propylene diene monomer

**DOI:** 10.1038/s41598-021-83692-1

**Published:** 2021-03-01

**Authors:** Jae Kap Jung, In Gyoo Kim, Ki Soo Chung, Un Bong Baek

**Affiliations:** 1 Center for Materials and Energy Measurement, Korea Research Institute of Standards and Science, Daejeon, 34113 Korea; 2 ICT Creative Research Laboratory, Electronics and Telecommunications Research Institute, Daejeon, 34129 Korea; 3 Department of Physics, The Research Institute of Natural Science, Gyeongsang National University, Jinju, 52828 Korea

**Keywords:** Techniques and instrumentation, Physics

## Abstract

We established an ex-situ technique for evaluating hydrogen gas permeability by thermal desorption analysis (TDA) gas chromatography (GC) and by self-developed diffusion analysis software. Absorbed hydrogen mass in rubber, related to the GC-peak area, is recorded as a function of elapsed time after decompressing the hydrogen under high-pressure. From the charging amount (C_H0_) and diffusivity (D) obtained by the developed diffusion analysis program, the solubility(S) and permeability(P) is evaluated via Henry’s law and P = SD, respectively. The techniques were applied to ethylene propylene diene monomer (EPDM) rubber, sealing material candidates in hydrogen infrastructures. EPDM sample mixed with carbon black fillers showed dual hydrogen diffusion behaviors, whereas EPDM sample without carbon black showed a single hydrogen diffusion behavior. There was no appreciable pressure or size dependence on D, S and P. P are consistent with that measured by different researcher within the expanded uncertainty.

## Introduction

Hydrogen gas can penetrate and diffuse into materials because hydrogen molecules are very small and lightweight. Penetrated hydrogen can cause material embrittlement, fractures, and extensive damage. Hydrogen embrittlement is a significant issue that threatens the safety of metals in facilities using hydrogen, and therefore, multiple studies have been conducted on the embrittlement of metals in hydrogen environments^1–5^.

Compared to metals, hydrogen can penetrate organic materials such as plastics and rubbers more easily and cause changes in their physical properties^6^. Rubber O-rings are used to seal high-pressure (HP) hydrogen gas in hydrogen stations and hydrogen fuel cell vehicles. For example, polymer rubbers are applied for compressors, dispensing hoses, threaded connectors, and valves in HP hydrogen infrastructures, including valves that control the discharge of hydrogen gas from gas storage tanks^7–9^. These rubbers are key components to ensure safety and gas tightness by preventing hydrogen leakage and withstanding harsh environments in contact with hydrogen, such as a HP of 90 MPa and a wide temperature range of − 40–90 ℃. O-ring rubber materials contain polymers such as ethylene propylene diene monomer (EPDM), nitrile butadiene rubber (NBR), and fluoroelastomer (FKM).

To discover candidates for suitable sealing materials to reduce the hydrogen permeability and withstand harsh hydrogen environments, measurements of hydrogen transport properties are important, and an elaborate evaluation method of the permeability characteristics that explains hydrogen penetration and diffusion in rubber is required. Furthermore, the international standard UN-GTR 13 proposes that materials should comply with SAE J2579 for the conformity assessment of metals and nonmetallic materials used in contact with hydrogen. In the case of SAE J2579, the CSA/ANSI CHMC 2 standard updated for polymeric materials is currently under development^10^. Here, it is also necessary to develop techniques to evaluate the permeation characteristics of hydrogen to establish national standards for the hydrogen economy.

Thermal desorption system (TDS) are useful for the quantitative analysis of hydrogen behaviors in rubber samples. The Nishimura group used this method to measure only hydrogen solubility and compared the values with those taken by the permeation method to verify the TDS^11^. The differential pressure method^12,13^ according to the ASTM D143 standard was utilized for measuring permeation and diffusivity in a specimen plate placed between two chambers at the feed side and permeation side. This method is effective for an in situ measurement of the permeability by monitoring the total average pressure. However, securing the traceability of the measurand, obtaining a precise determination of the dimensions of the specimen under HP and performing an uncertainty analysis were further required to obtain reliable measurements.

In this study, we established an ex situ thermal desorption analysis-gas chromatography (TDA-GC) method with the application of a self-developed diffusion analysis program to evaluate the entire permeation characteristics such as solubility, diffusivity and permeability by applying the solution of Fick’s second laws of diffusion to the amount of hydrogen gas released from a rubber sample after HP hydrogen charging and decompression. This method was first applied to EPDM rubber, which are sealing material candidates for hydrogen gas in hydrogen infrastructures. The study is focus to clarify the permeation characteristics for variations in pressure and sample size. To verify the consistency of the measured results for the diffusivity, solubility and permeability in the EPDM rubber, we compared the results with those obtained by the differential method and group. The overall procedure for obtaining the permeation properties from GC measurements is detail described quantitatively together with the uncertainty evaluation. The features of this method are briefly described in the conclusion.

## Diffusion in cylindrical solids

In a non-steady state, the diffusion flux and the concentration of diffusing particles change over time, so Fick’s second law of diffusion^14^ at cylindrical coordinate is written as follows.

1 $$ \frac{\partial C}{{\partial t}} = D\left( {\frac{\partial C}{{\partial r^{2} }} + \frac{1}{r} \frac{\partial C}{{\partial r}} + \frac{{\partial^{2} C}}{{\partial z^{2} }}} \right) $$

where *D* is the constant hydrogen diffusion coefficient and *C* is the hydrogen concentration with appropriate initial and boundary conditions; initially a constantly uniform hydrogen concentration is maintained, and the cylindrical surfaces are kept at a constant concentration. Hydrogen dissolved in rubber under HP is released from the rubber due to the pressure difference when the pressure is decompressed. With the assumption that the hydrogen release profile satisfies the Fickian diffusion process, the change in the amount of residual hydrogen inside the samples over time is as shown in Eq. (2)^15^.

2 $$ C_{H,R} \left( t \right) = \frac{32}{{\pi^{2} }} \times C_{H0} \times \left[ {\mathop \sum \limits_{n = 0}^{\infty } \frac{{exp\left\{ { - \left( {2n + 1} \right)^{2} \pi^{2} Dt/l^{2} } \right\}}}{{\left( {2n + 1} \right)^{2} }}} \right] \times \left[ {\mathop \sum \limits_{n = 1}^{\infty } \frac{{exp\left\{ { - D\beta_{n}^{2} t/\rho^{2} } \right\}}}{{\beta_{n}^{2} }}} \right] $$

This is the solution of the law of diffusion in Eq. (1), i. e., Fick’s second law, in the case of cylindrical samples. In Eq. (2), $C_{H,R} \left( t \right)$ is the amount of residual hydrogen at time $t$, as hydrogen is uniformly distributed in the cylindrical rubber and then diffuses into a vacuum after decompression. In Eq. (2), $l$ is the thickness of the cylindrical rubber sample, $\rho$ the radius, and $\beta_{n}$ is the root of the zero-order Bessel function. The diffusivity of hydrogen, $D$, and total charged amount of hydrogen, $C_{HO}$, is obtained by applying $C_{H,R} \left( t \right)$ data to Eq. (2).

On the right side of Eq. (2), the function of the former bracket becomes $$\frac{{\pi^{2} }}{8}$$, and the latter becomes $$\frac{1}{4}$$ when $$t = 0$$. Therefore, the product of the two brackets becomes $$\frac{{\pi^{2} }}{32}$$, and then $$\frac{32}{{\pi^{2} }}$$ is inserted into the Eq. (2) so that the residual amount becomes $$C_{HO}$$ when $$ t = 0$$. The derivative of Eq. (2) at $$ t = 0$$, that is $$\frac{{dC_{H,R} \left( {t = 0} \right)}}{dt}$$ =  − ∞, and this means that the initial escape rate of hydrogen is extremely high. This phenomenon is due to an extreme distribution of hydrogen caused by the discontinuous pressure difference between the HP inside the rubber and the vacuum on the outside after decompression. The first two or three terms of two summations in Eq. (2) contribute to the $$C_{H,R} \left( t \right)$$ value only when $$t$$ is large enough (above 1 s). However, in the case of a short time, when $$t$$ is less than $$t = 0.1 s$$, Eq. (2) cannot converge. Therefore, at least five or more terms are required, and a dedicated program is necessary for the precise analysis. We thus developed a diffusion analysis program using Visual Studio, which allows us to calculate the *D* and the C_H0_, including up to the 50^th^ term of both the first- and second-brackets of Eq. (2). Using the calculated results, we can obtain $$S$$ by Henry’s law of the $$S = \frac{C}{p}$$ relation and $$P$$ by the relation of the $$P = DS$$.

## TDA-GC processes

**Figure 1:**
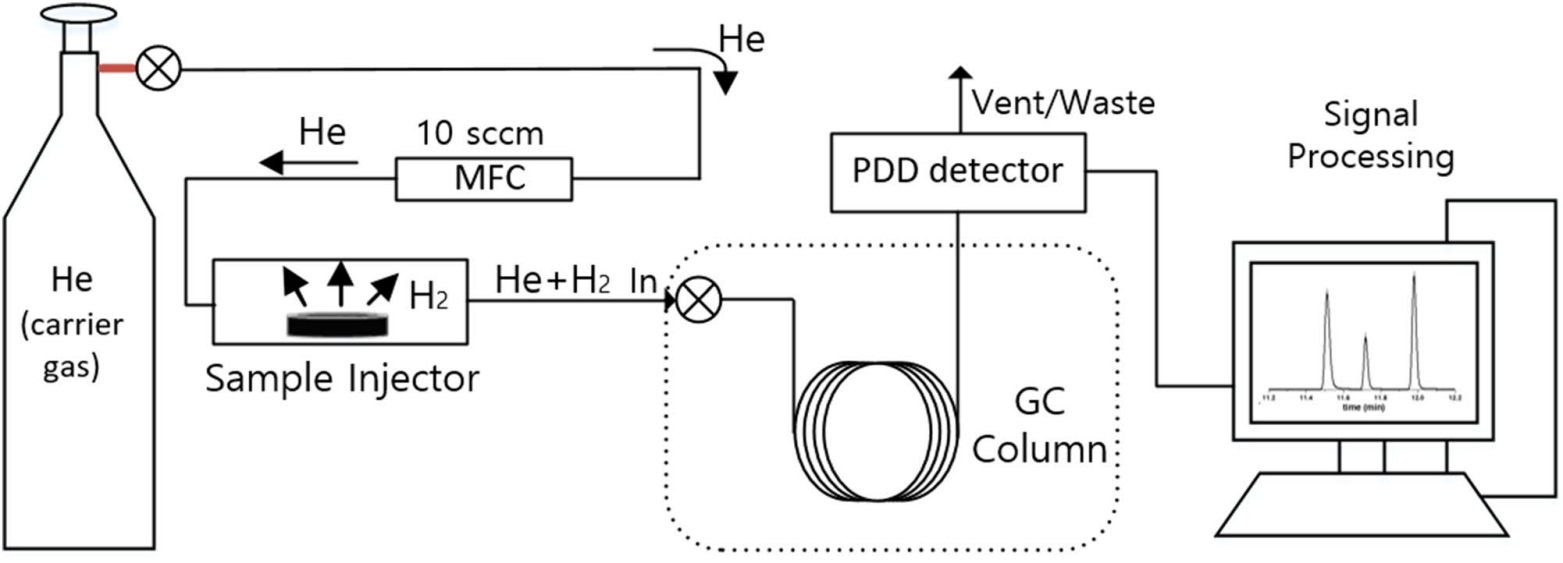
Configuration of the TDA-GC setup.

TDA-GC is used to separate and measure various types of gases from mixed gas components discharged from a sample^16,17^. This qualitatively and quantitatively analyzes the corresponding gas by measuring the position and area of the separated GC signal. Figure 1 shows the configuration of a TDA-GC setup. The flow rate of helium gas (carrier gas) is controlled through a mass flow controller (MFC), and the sample to be analyzed is located in a quartz tube (sample injector). The outgassing from the sample is mixed with the carrier gas and is sent to the GC column through the injector. The pulsed discharge detector (PDD) in GC converts the separated gas components into electrical signals that are sent to signal processing devices such as PCs to obtain peaks corresponding to the separated gas components. As a result, the molar concentration of gas released from the sample can be measured, and then the permeation characteristics of the gas can be analyzed.

**Figure 2:**
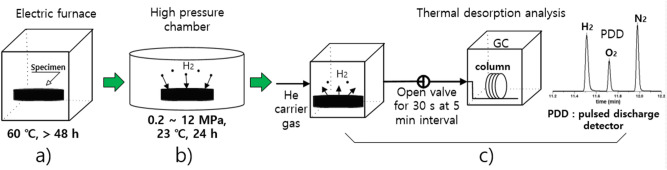
Schematic diagram of the overall TDA-GC measurement procedure. (**a**) heat treatment is performed at 60 ℃ for at least 48 h to minimize outgassing from the rubber, (**b**) hydrogen charging at room temperature and the desired pressure for 24 h. (**c**) the pressure is lowered to atmospheric pressure by opening the exhaust valve after the charging process, then, the specimen is removed to start the GC measurement.

Figure 2 shows the overall TDA-GC procedure before analyzing rubber samples. First, heat treatment is performed at 60 ℃ for at least 48 h, as recommended in CHMC 2^10^, to minimize outgassing from the rubber (Fig. 2a), followed by hydrogen charging at room temperature and at the desired pressure for 24 h (Fig. 2b). The charging for 24 h is found to be sufficient to arrive the equilibrium by measuring the penetrating amount (C_H0_) of hydrogen in sample as a function of charging time. The pressure is lowered to atmospheric pressure by opening the exhaust valve of high pressure chamber with the rate of approximately 1 MPa/s. After decompression, the specimen is removed from the chamber to start the GC measurement (Fig. 2c). The elapsed time is recorded from the moment (t = 0) at which the HP hydrogen gas chamber is reduced to atmospheric pressure. Thus inevitable time lag (6 min) between the decompression and the start of GC measurement exists. As a result, a comparatively large volume of hydrogen was already released from the specimen before the GC measurement tests, because of time delay.

The detailed processes of hydrogen permeation property analyses in the rubber from the TDA-GC measurements are as follows.

**Figure 3:**
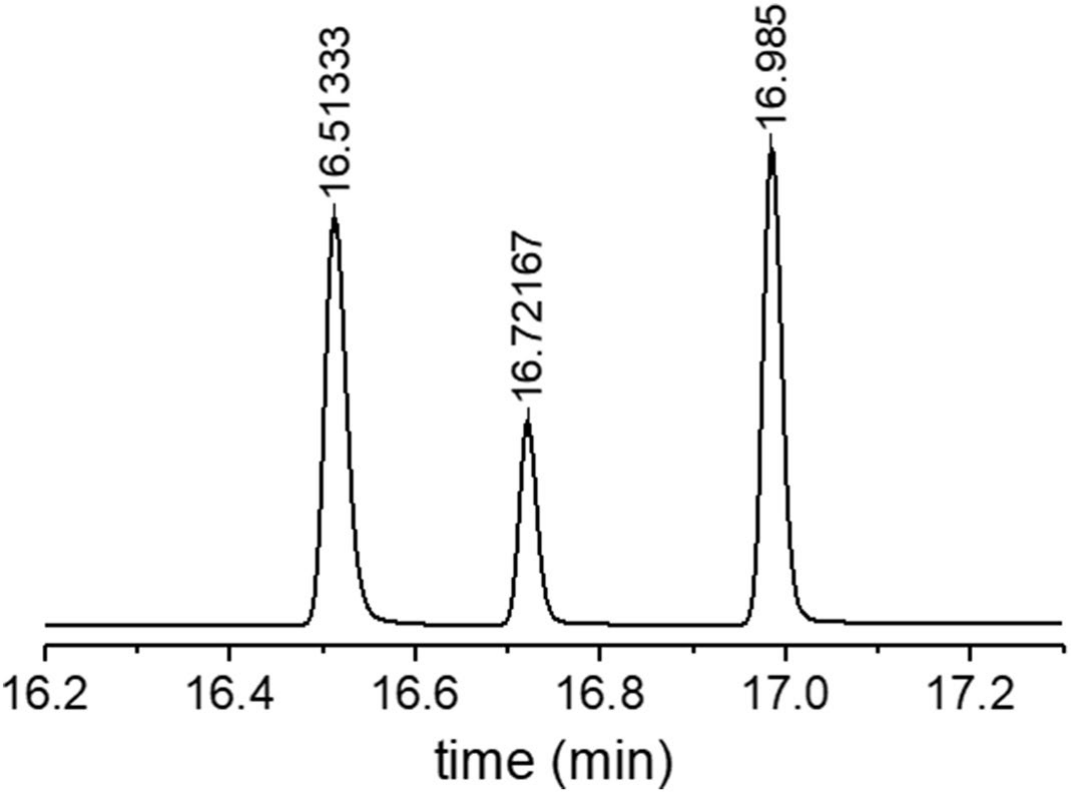
GC spectra measured by injection after 15 min from the start of measurement by loading in the GC specimen chamber. The results obtained after decompressing an EPDM sample (cylindrical shape, diameter: 4.1 mm, thickness: 2.6 mm) filled with HP hydrogen for 24 h in a container at 4.85 MPa. The GC signals of hydrogen, oxygen, and nitrogen molecules are shown from left to right. Oxygen and nitrogen signals come from the air not emitted by the rubber because of the contact with air environment during sample moving.

**Figure 4:**
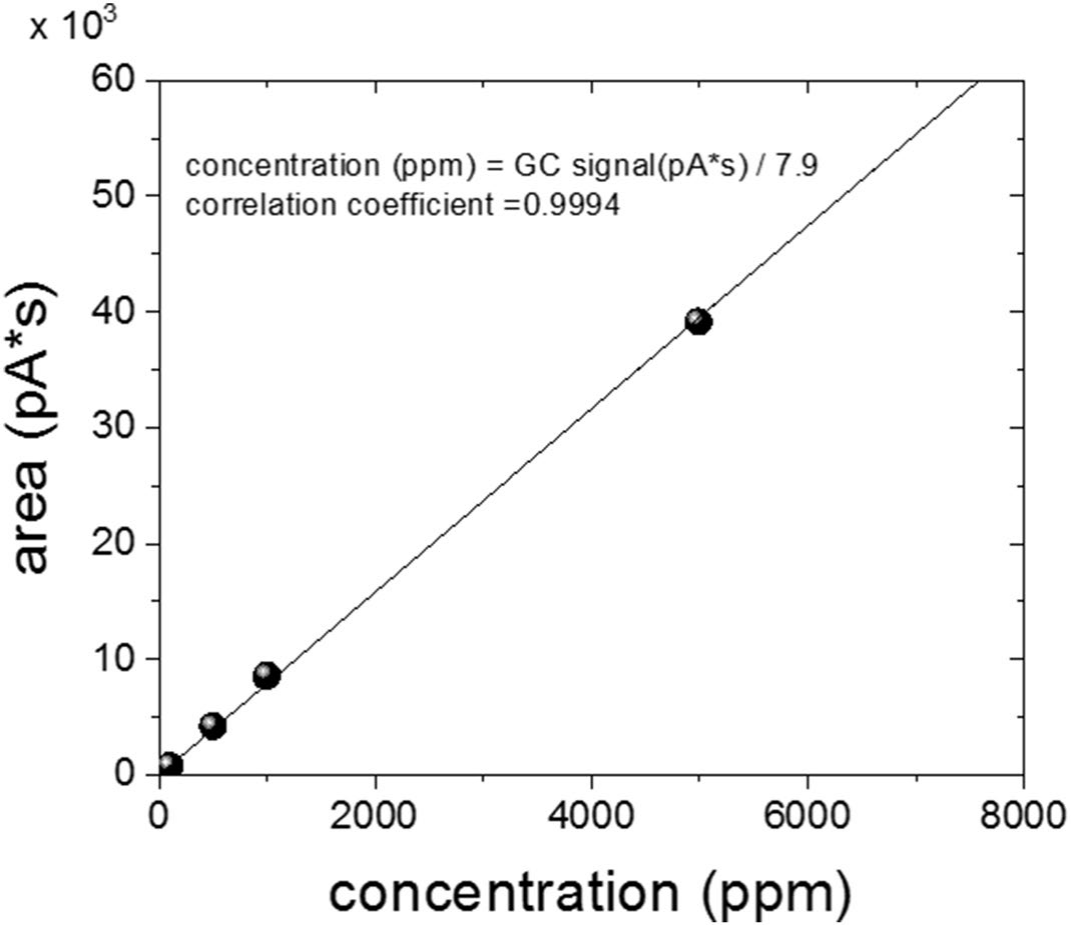
Calibration curve obtained for standard hydrogen gases with hydrogen concentrations of 100 ppm, 500 ppm, 1000 ppm, and 4989 ppm with helium gas as the balance. (correlation coefficient: 0.9994).

(i) After decompression, the sample was placed in a quartz tube with an inner diameter of 14 mm and a length of 100 mm connected to the GC system (Agilent 7890A GC system). Then, the hydrogen gas that was diffused and released from the sample was introduced into a capillary GC column (inner diameter of 0.32 mm) with helium gas at a flow rate of 10 sccm, and the hydrogen gas that was separated in the column was detected by a PDD (Fig. 1).
(ii) The oven temperature of the GC column was set to 100 °C, and the temperature of the detector was set to 120 °C. At different time intervals, the gas released from the sample was injected by opening the valve for 0.5 min at 0.1, 3, 5, 10….0.990 min for a total of 16.5 h. The example of a hydrogen gas signal, which appeared approximately 15 min after each injection, corresponds to the peak on the left side in Fig. 3.
(iii) To determine the molar concentrations corresponding to the area with units of pA·s for the hydrogen peak of the transformed electrical signal, we produced a calibration curve, as shown in Fig. 4 using standard hydrogen gas with known concentrations of 100 ppm, 500 ppm, 1000 ppm, and 4989 ppm. Thus, the area of the hydrogen signal obtained with units of pA·s is transformed to the molar concentration of hydrogen using the linear slope (pA·s/7.9) in the calibration curve.
(iv) To obtain the absolute mass of hydrogen charged in a sample from the GC measurement, we need to know the flow rate of the gas sample corresponding to one GC signal as well as the volume, temperature, and pressure of the sample taken from the sample loop. The released gas is injected into the GC system at a flow rate of 10 sccm at room temperature. We can quantify the mass of hydrogen and the number of moles corresponding to a GC signal are as follows.

By assuming an ideal gas equation $\left( {PV = nRT} \right)$ under a constant volume and temperature, the total number of moles (*n*) of the mixed gas is calculated by substituting the volume of the sample collected in the sample loop sent to the GC column (V = 0.25 cc = 2.5 × 10^–7^ m^3^) and the gas constant (R = 8.20544 × 10^–5^ m^3^·atm/mol·K) at 1 atm and 298 K as follows:

3 $$ n = \frac{{1\;{\text{atm}} \cdot 2.5 \times 10^{ - 7} \;{\text{m}}^{3} }}{{\left( {8.20544 \times 10^{ - 5} {\text{m}}^{3} \cdot \frac{{{\text{atm}}}}{{{\text{mol}} \cdot {\text{K}}}} \cdot 298\;{\text{K}}} \right)}} \cong 1.0224 \times 10^{ - 5} \;{\text{mol}} $$

If the GC-measured concentration, i. e., the molar concentration of hydrogen converted by Fig. 4, is *C* ppm, the number of moles of hydrogen gas in the mixed gas can be obtained from the equation below:

4 $$ n_{{H_{2} }} = {\text{Cppm}} \cdot \left( {n_{{H_{2} }} + n_{He} } \right) = {\text{Cppm}} \cdot n = 1.0224 \times 10^{ - 11} \;{\text{C}}\;{\text{mol}} $$

where $n_{{{\text{H}}_{2} }}$ and $n_{{{\text{He}}}}$ are the number of moles of hydrogen and helium, respectively. Using Eq. (4), we can obtain the total number of moles of charged hydrogen by the GC measurements over time after charging the hydrogen under HP and estimate the hydrogen mass.

**Figure 5:**
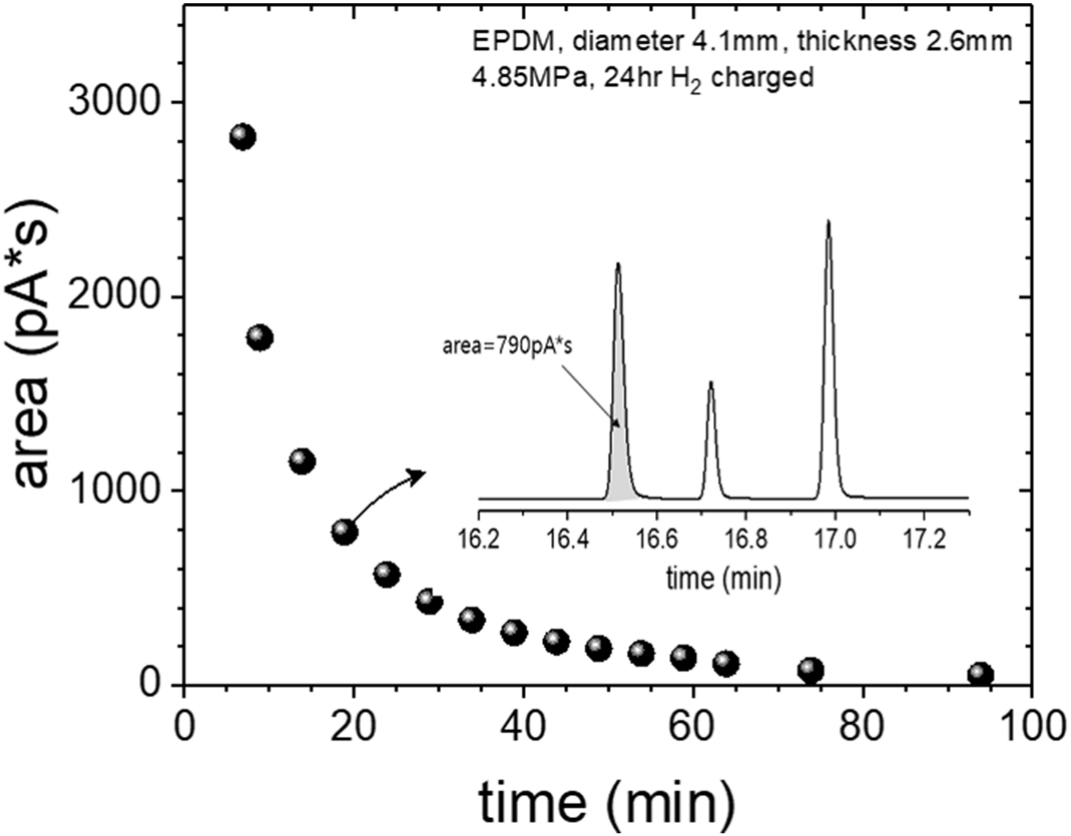
Measured GC signal intensity (area) against the elapsed time after decompression (sample: hydrogen charged EPDM for 24 h in 4.85 MPa pressure).

For instance, the process for obtaining the hydrogen content charged in an EPDM sample with a diameter of 4.1 mm, a thickness of 2.6 mm, and a mass of 0.0424 g exposed to HP (4.85 MPa) hydrogen gas for 24 h is as follows. The intensity of the GC signals decreases as time goes after decompression as shown in Fig. 5. In the figure, the inset shows a set of typical GC signals originated from one injection at elapsed time of 15 min, and the area of the first one, which is a H_2_ signal, was taken as a data.

By using calibration curve in Fig. 4, the GC intensity data in Fig. 5 can be converted to the molar concentration of hydrogen gas, and further to the mass ppm concentration with respect to the mass of EPDM sample as follows. Each molar concentration obtained applying the calibration data is the hydrogen concentration (*C*) in the gas contained in the sample within the sample loop volume (2.5 × 10^–7^ m^3^). Once this value is converted to the number of moles using Eq. (4), it can be converted to a mass by multiplying by the molar mass of 2.018 g/mol. Since it takes 1.5 s to fill the 0.25 cc sample loop at a flow rate of 10 sccm, we obtain a value in mass concentration per second for each GC signal dividing the mass values by 1.5 s, and by the mass of the sample, 0.0424 g. The converted value indicates the mass concentration of hydrogen released per second for each GC measurement for one injection in units of wt·ppm/s.

The process above can be summarized into the following equation:

5 $$ n = \frac{{P \cdot V_{SL} }}{R \cdot T} $$

6 $$ C_{{{\text{mass}}}} \left( {\frac{{{\text{wt}} \cdot {\text{ppm}}}}{{\text{s}}}} \right) = C_{{{\text{mol}}}} \left( {{\text{mol}} \cdot {\text{ppm}}} \right) \times \left\{ {\frac{{n\left( {{\text{mol}}} \right) \cdot m_{{H_{2} }} \left( {\frac{{\text{g}}}{{{\text{mol}}}}} \right)}}{{m_{{{\text{sample}}}} \left( {\text{g}} \right)}}} \right\} \times \left\{ {\frac{{v_{He} \left( {\frac{{m^{3} }}{s}} \right)}}{{V_{SL} \left( {m^{3} } \right)}}} \right\} = \frac{{C_{mol} \cdot P \cdot m_{{H_{2} }} \cdot v_{He} }}{{m_{sample} \cdot R \cdot T}} $$

where $C_{{{\text{mass}}}} \left( {\frac{{{\text{wt}} \cdot {\text{ppm}}}}{{\text{s}}}} \right)$ is the mass concentration of hydrogen to the sample mass measured per unit time, $m_{{H_{2} }}$ is the molar mass of hydrogen, $V_{SL}$ is the volume of the gas specimen taken for a single injection in the sample loop of the GC system (0.25 cc), $v_{He}$ is the flow rate of the carrier gas (10 sccm), and $m_{{{\text{sample}}}}$ is the mass of the sample. Here, $\frac{{V_{SL} }}{{v_{He} }}$ is the time it takes for the gas sample to fill the sample loop volume located in the GC valve (1.5 s). Equation (6) calculates the mass concentration of hydrogen independent of the sample loop volume (0.25 cc). Under the experimental conditions of 1 atm, 298 K, and a flow rate of the helium carrier gas of 10 sccm (≈1.67 × 10^–7^ m^3^/s), the equation above is simplified to Eq. (7).

7 $$ \begin{aligned} C_{mass} \left( {\frac{{{\text{wt}} \cdot {\text{ppm}}}}{{\text{s}}}} \right) &=  \frac{{C_{mol} \left( {{\text{mol}} \cdot {\text{ppm}}} \right) \times \left( {1\;{\text{atm}}} \right) \times \left( {2.018\frac{{\text{g}}}{{{\text{mol}}}}} \right) \times \left( {1.67 \times 10^{ - 7} \;\frac{{{\text{m}}^{3} }}{{\text{s}}}} \right)}}{{\left( {m_{sample} } \right) \times \left( {8.20544 \times 10^{ - 5} {\text{m}}^{3} \cdot \frac{{{\text{atm}}}}{{{\text{mol}} \cdot {\text{K}}}}} \right) \times \left( {298\;{\text{K}}} \right)}} \\ &\cong  1.378 \times 10^{ - 5} \frac{{C_{mol} \left( {{\text{mol}} \cdot {\text{ppm}}} \right)}}{{m_{sample} \left( {\text{g}} \right)}} \\ \end{aligned} $$

**Figure 6:**
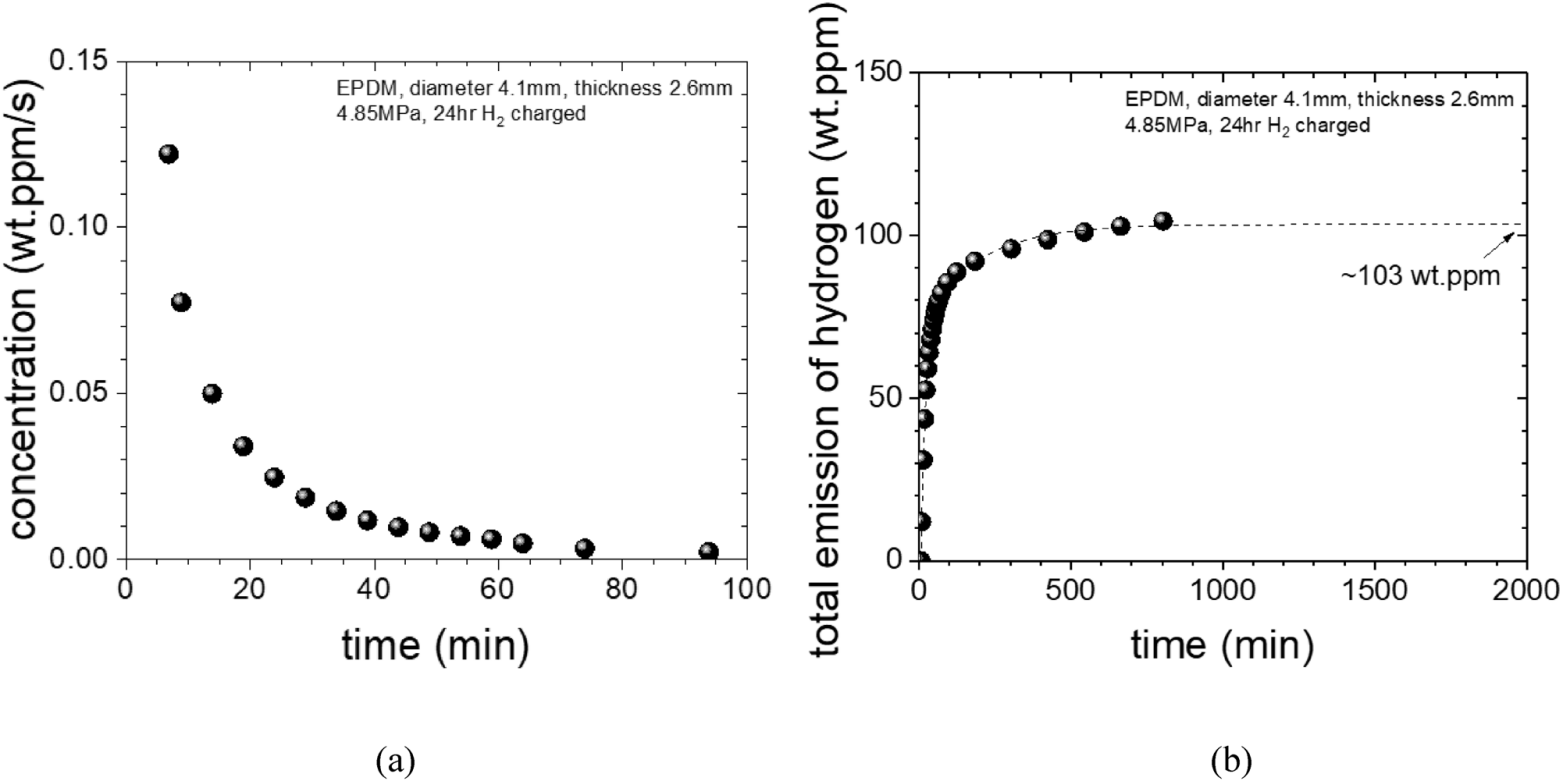
Time-dependent hydrogen concentration curve of an EPDM sample expressed in mass concentration versus time. (**a**) is transformed into mass concentration of hydrogen per second from the GC data in Fig. 5, (**b**) is the integration of the curve for (**a**) it gives us the saturation value, i.e. the total amount of the hydrogen charged into the EPDM sample. In this example, the extrapolated total hydrogen contents is 103 wt.ppm.

As a result, the GC data in Fig. 5 is transformed into mass concentration of hydrogen per second, as shown in Fig. 6a. The integration of the curve [Fig. 6a] against time it gives us the information about the saturation value, i.e. the amount of the hydrogen charged into the EPDM sample as shown in Fig. 6b. In this example, the extrapolated hydrogen contents is 103 wt∙ppm, which is saturated value at infinite long time, which is obtained from the measurement of t = 6 min due to time lag. Thus the amount of hydrogen emitted from t = 0 min to t = 6 min are missing. The diffusion analysis program is applied to restore the lost hydrogen content and total charging amount (C_H0_).

**Figure 7:**
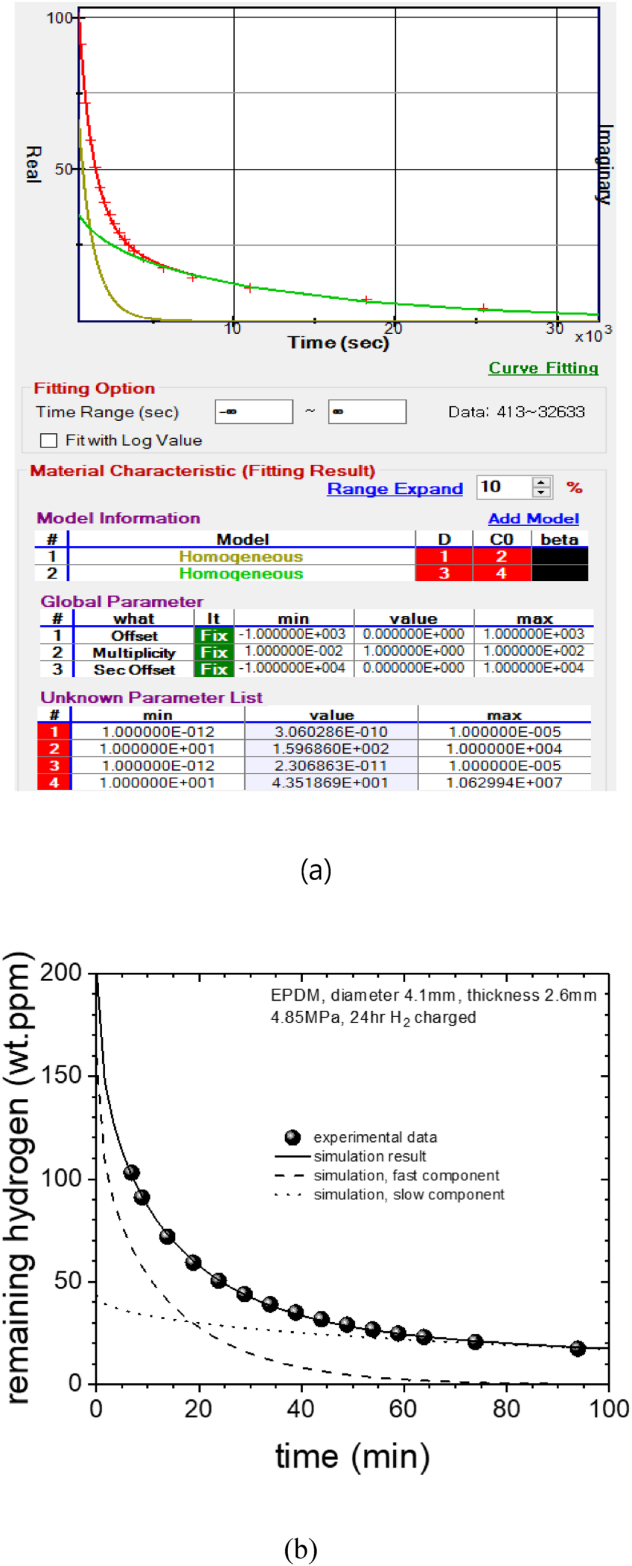
(**a**) The image of analysis program showing two kinds of diffusion behaviors. The parameters, D and C_H0_ are the diffusivity and the residual hydrogen content, respectively. (**b)** Experimental data (symbols) of residual hydrogen concentration and simulation results (dashed- and dotted-lines).

By substracting the emission values from the estimated charged value(103 wt ppm), we obtain remaining hydrogen contents which correspond to C_H,R_ in Eq. (2), and further obtain the *D* and *C*_H0_ through the diffusion analysis program by the least square method. For this purpose, we developed an analyzing program which can treat cylindrical shaped samples with different sizes including up to 50th term. The working window of the program looks like as shown in Fig. 7a. The analysis result show that there are two components of diffusion characteristics, i.e. slow and fast, and the experimental data are well fit with the sum of these two components as shown in Fig. 7b. For the above example, the *D* and the *C*_H0_ are as follow: D_fast_ ≈ 3.06 × 10^–10^ m^2^/s, C_H0-fast_ ≈ 160 wt·ppm and D_slow_ ≈ 2.31 × 10^–11^ m^2^/s, C_H0-slow_ ≈ 43.5 wt·ppm. In summary, as a result of charging hydrogen at 4.85 MPa into a cylindrical EPDM sample (diameter: 4.1 mm, thickness: 2.6 mm), we found that a total amount of 203.5 wt·ppm of hydrogen penetrated the EPDM. This value C_H0_ corresponds the value on y-axis at t = 0, obtained by extrapolating the fitted line with analysis program (Fig. 7).

By the reason discussed in the next section, the component with a large (fast) diffusion coefficient is due to the hydrogen absorbed in the main macromolecular polymer that constitutes the rubber, and the small (slow) component is due to hydrogen absorbed in the carbon black (CB) filler, which is the same interpretation as the result given by Nishimura group^11^.

## Results and discussion

When manufacturing EPDM rubber, large quantities of CB or silica are added as reinforcing agents, additives, and fillers to improve the thermal, electrical, and physical properties. Carbon black of 34% was included as a filler during fabrication of the EPDM specimen. Fast Extrusion Furnace (FEF) carbon black (N550) with particle size of 40 nm to 49 nm is used. The chemical compositions and properties or function of the EPDM rubbers are shown in Table 1. The CB in our sample used as a filler shows differences in hydrogen gas behavior after decompression by differences in the hydrogen absorption and permeation characteristics between the filler and the rubber matrix. We can separate the different behaviors by analyzing the amount of residual hydrogen over time as explained above.

**Table 1:** Chemical compositions according to function and related properties of EPDM specimen.

Function or property	EPDM
Polymer	EPDM (58)
Filler-reinforcing	Carbon black (34)
Curing agent	Zinc oxide (3) Dicumyl peroxide (5)
Relative crystallinity^+^ (%)	18
Density (g/cm^3^)	1.15

*Numbers in ( ) are weight ratios in %; ^+^ relative crystallinity (%) = 100–amorphous (%).

**Figure 8:**
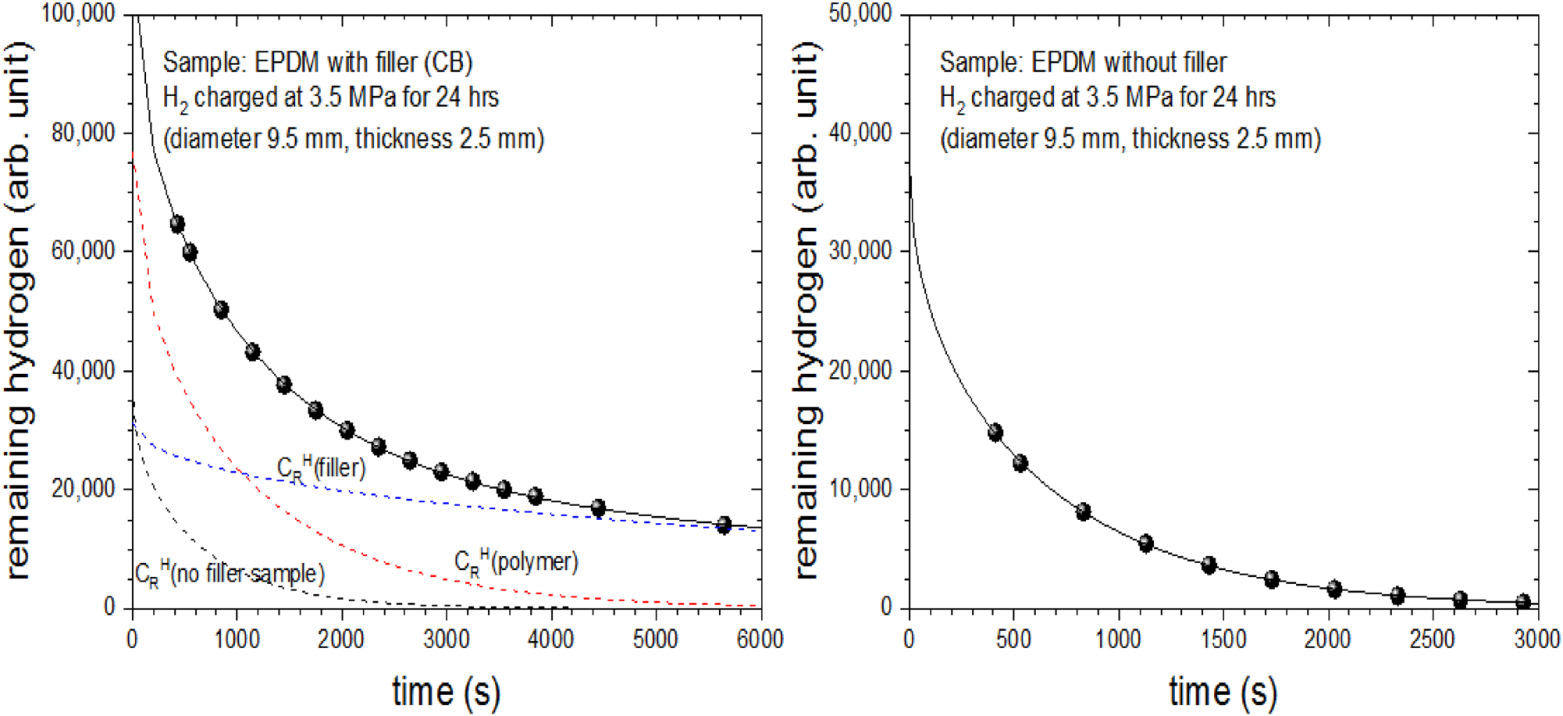
The different diffusion behaviors of residual hydrogen contents over elapsed time in the EPDM sample with CB filler (left) and without CB filler (right).

Figure 8 show the amount of residual hydrogen over elapsed time for EPDM samples with and without CB fillers, where the filled circles are the experimental data. On the left side of Fig. 8, the black solid line is the fitted sum of the blue dotted line and red dotted line, which are the two simulation results using the diffusion analysis program shown in Fig. 7 using two Eqs. (2). The standard deviation in the fit results between the experimental data and diffusion model is found to be less than 1%, implying two same equations with different C_H0_ and D are well applied for data simulation. The EPDM sample with CB filler is analyzed as containing two types of hydrogen gas with different diffusivities. The red dotted line is interpreted as the fast diffusion of the sample according to the hydrogen behavior in the polymer network [C_R_^H^(polymer)], while the blue dotted line is the relatively slow diffusion due to the hydrogen behavior in the filler [C_R_^H^(filler)]. As shown in Fig. 8, the permeation behavior of hydrogen gas in EPDM was interpreted as arising from two components of hydrogen absorbed in the rubber matrix and filler. Moreover, in the EPDM sample without CB filler, the results simulated(black solid line on right side of Fig. 8) with a diffusion model in a single term of Eq. (2) were consistent with the experimental data. The diffusion coefficients of filler-free samples of EPDM appeared faster than any of the other filler-added samples, implying that the hydrogen molecules adsorbed in the rubber matrix show faster diffusion behaviors than those in the filler.

**Figure 9:**
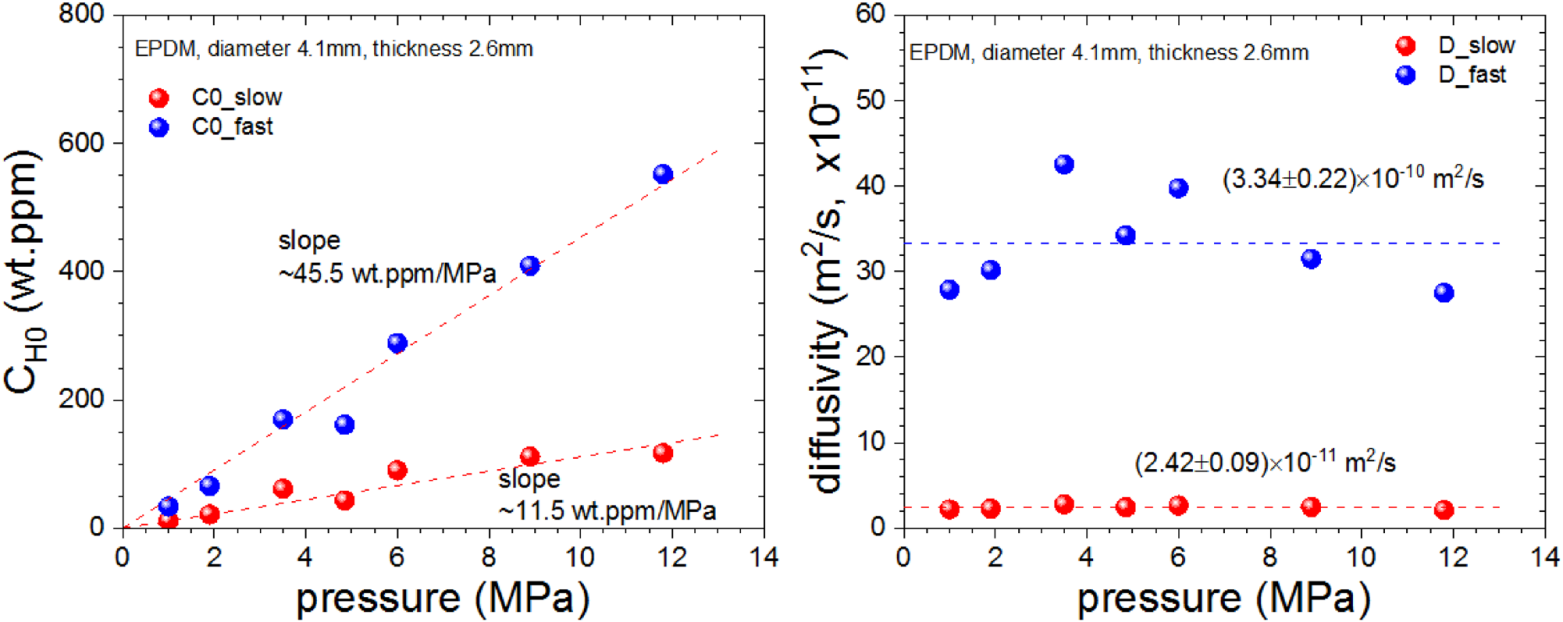
The content and diffusivity of the EPDM sample with CB filler versus pressure.

To examine the permeation characteristics of the EPDM rubber samples, we charged hydrogen for 24 h in the pressure range from 0.2 to 12 MPa to measure and analyze the release of hydrogen over time with both GC and the developed diffusion analysis program after decompression. Figure 9 also show the dual hydrogen behaviors (fast and slow) for both the hydrogen content and diffusivity data with respect to the pressure in the cylindrical EPDM samples with CB filler of a radius of 4.1 mm and a thickness of 2.6 mm. The hydrogen content data approximately follow Henry’s law, and the hydrogen solubility (*S*) of each sample was obtained from the slope of the hydrogen content charged in the sample according to pressure. Since the observed diffusivity in the sample on the right sides of Fig. 9 does not show pressure-dependent behavior, its representative value for various pressures was taken as the average value. Thus, the permeability (*P*) was obtained by calculating the average diffusivity (*D*_*ave*_) at each charging pressure through a program simulation and by multiplying it by solubility (*S*).

The uncertainty of technique used is evaluated following the guide to the “Expression of Uncertainty in Measurement” (GUM)^18^. The uncertainty source and expanded uncertainty of measurement for hydrogen permeation parameters are summarized in Table 2. The contributions to the uncertainty in the solubility and diffusivity measurement are primarily due to the stability of measurements evaluated by repeated measurements, the linear drift of TDA-GC, the uneven diameter and thickness of the sample, standard deviation between data and Eq. (2), the accuracy of the manometer, the resolution of the analog manometer, and the temperature variation in laboratory. The type A uncertainty resulting from repeated measurements is obtained by 3 times measurements.

**Table 2:** Summary of uncertainty source and expanded uncertainty for permeation parameters of hydrogen.

Source of uncertainty	Value (%)
Repeated measurements	5.7
Linear drift of TDA-GC	3.0
Uneven sample volume	2.9
Standard deviation between data and Eq. (4)	1.2
Accuracy of manometer	0.6
Resolution of analog manometer	1.0
Variation of temperature	1.0
Combined standard uncertainty, *u*_c_	7.3
Coverage factor, *k*	2.1
Expanded uncertainty, *U* = *ku*_c_	15.4

The all type B uncertainty contributions, except of manometer resolution, are obtained by dividing a factor of $\sqrt 3$ with an assumption of a rectangular distribution. We measured the linearity of GC three times for three months using He gas and standard hydrogen gas with concentrations of 100 ppm, 500 ppm, 1000 ppm, and 4989 ppm. The maximum deviation of the linearity reached 5.2%. Therefore, the type B uncertainty due to the drift can be obtained. After charging the sample, the maximum deviation for measuring the diameter and thickness of the sample using a Vernier caliper reached 5.0%. Thus the type B uncertainty due to uneven sample volume is acquired.

The standard deviation between the data for the amount of hydrogen versus time and the fit using Eq. (2) is within 2%, depending on the samples. Considering that the maximum deviation is 2%, the type B uncertainty can be obtained. The accuracy of manometer is 1%, which corresponds to GRADE A. Thus the type B uncertainty estimated is 0.6%. When the pressure measured by an analog manometer is 10 MPa, the minimum scale is 0.5 MPa, which corresponds relative value of 5%. Therefore, the type B uncertainty by the resolution can be obtained as 1.0% by dividing the factor of $2\sqrt 6$ with a triangular probability distribution. The laboratory environment is maintained at constant temperature 25 ℃ with variation of ± 2 ℃. The type B uncertainty due to the variations of temperature is estimated.

The combined standard uncertainty is represented as a root sum of squares of the individual uncertainty. The relative expanded uncertainty is obtained by assuming a normal distribution and multiplying the combined standard uncertainty by a coverage factor of 2.1 at a 95% confidence level. The estimated expanded uncertainty for solubility and diffusivity is not more than 15.4%.

**Figure 10:**
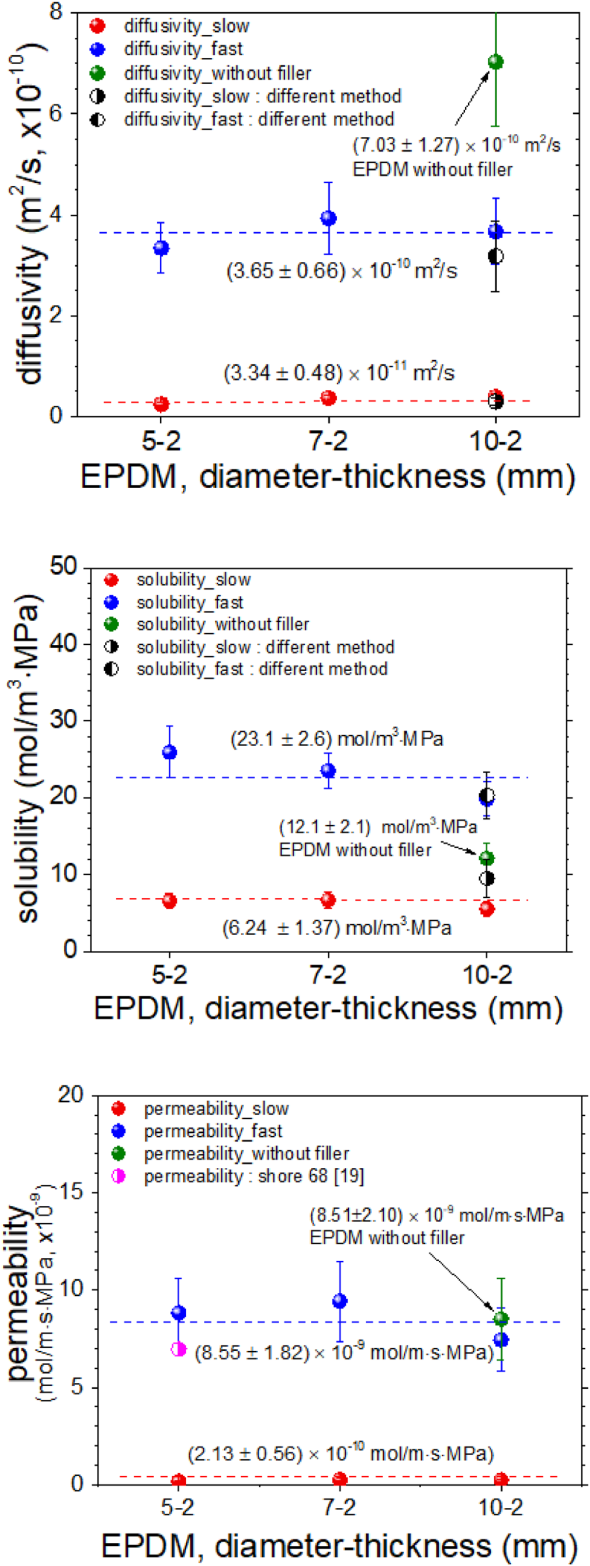
Permeation characteristics according to the size of the EPDM sample.

The permeation characteristics of the EPDM sample is shown in Fig. 10, together with those obtained with different diameters of approximately 7 mm and 10 mm, while maintaining the same thickness for each sample. The error bar indicates the expanded uncertainty estimated in this work. The top side of Fig. 10 shows the diffusivity of the fast and slow components of the EPDM sample together with those by volumetric analysis technique developed recently^19^. The diffusion coefficients obtained by TDA-GC in same sample are in satisfactory agreement with those by volumetric analysis technique. There was no significant difference in diffusivity according to the sample diameter within the uncertainty level. The two diffusivities of the EPDM sample with filler was measured to be slower than that of EPDM without filler. The decrease of the diffusion coefficient of EPDM with filler may be related with the decrease of the free volume, the increase both the tortuosity and density by the filler loading.

In the middle of Fig. 10, the solubility of the two types of diffusivity were consistent within the uncertainty range regardless of the sample diameter, and more hydrogen was dissolved in the fast component than in the slow component. The total solubility of EPDM with filler was larger than that of EPDM without filler. The sorption of hydrogen by CB is considered to be one of reasons for the increase in solubility. The Fig. 10 shows the solubility in same sample obtained by this method coincide with those by volumetric analysis technique within expanded uncertainty. Furthermore, the total solubility of our EPDM (~ 29 mol/m^3^ MPa), which is comparable to that (~ 34 mol/m^3^ MPa) in EPDM with CB 25 obtained by other group^11^.

There was also no dependence on permeability as a function of diameter, as shown in the permeability results on the bottom side of Fig. 10. The permeability of hydrogen with fast diffusivity is greater than that of hydrogen with slow diffusivity, as expected. As a result of comparing the permeability of our EPDM samples with EPDM of shore hardness of 68 by Pauly group^20^, we can see that the values in^20^ were similar to those obtained for both permeability of the fast component for the EPDM with filler and permeability for the EPDM without filler obtained in our study within the uncertainty.

As shown in the diffusivity results on the top of Fig. 10, the diffusivities of the EPDM samples without fillers are approximately twice as large as those of the fast components of EPDM with fillers. Based on this result, we can infer the following. First, the fast component shows the permeation characteristics of hydrogen adsorbed onto the parent component of the rubber. Second, the slow component shows the permeation characteristics of hydrogen adsorbed to the filler. These findings are consistent with the analysis in the study by the Nishimura group^11^.

## Conclusions

We established a method to evaluate the permeation characteristics of hydrogen by analyzing the amount of hydrogen gas released after decompression using the TDA-GC method, together with uncertainty evaluation. By considering the high-order terms up to the 50th summation terms in Eq. (2), we developed a special diffusion analysis program to evaluate the total charged amount C_H0_ and diffusivity D. Without application of the dedicated program, especially C_H0_ value could not be determined precisely, because of time lag between the decompression and the start of TDA-GC measurement.

This is the first report to apply this technique to cylindrical EPDM samples to evaluate the full permeation characteristics of hydrogen gas according to changes in both pressure and sample size. The evaluation results for the EPDM showed that the permeation characteristics of hydrogen, that is, the measurement results of diffusivity, solubility, and permeability, were not significantly dependent on sample size. Hydrogen in the EPDM samples with filler was revealed to have two types of diffusion, namely, fast diffusion due to the hydrogen adsorbed in the polymer network and slow diffusion due to the hydrogen absorbed in the filler. Moreover, the results of measuring the charged amount under a pressure up to 12 MPa can be explained by Henry’s law, which confirmed that the content is primarily proportional to the pressure. Based on this result, we can obtain the solubility. The results of evaluating the permeation properties of the rubber were consistent with the results of different technique and prior studies within the uncertainty level, thereby validating the technique developed in this study.

TDA-GC is a sophisticated technique for observing hydrogen behaviors and can obtain the respective diffusivities by independently separating two or more behaviors from mixed hydrogen groups. GC was successfully used for the analysis of multicomponent gas permeation. This method can also quantify the amount of hydrogen gas charged in a small sample and measuring the absolute mass of hydrogen gas by using the standard gases traceable to national standards. Further research to reduce the Type A uncertainty with a good quality sample and inter comparison with different group is also required.
